# Warming Increases Pollen Lipid Concentration in an Invasive Thistle, with Minor Effects on the Associated Floral-Visitor Community

**DOI:** 10.3390/insects11010020

**Published:** 2019-12-25

**Authors:** Laura Russo, Joseph Keller, Anthony D. Vaudo, Christina M. Grozinger, Katriona Shea

**Affiliations:** 1 Department of Entomology and Plant Pathology, University of Tennessee, Institute of Agriculture, Knoxville, TN 37996, USA; 2 Department of Biology, Pennsylvania State University, State College, PA 16802, USA; jak573@psu.edu (J.K.); k-shea@psu.edu (K.S.); 3 Department of Entomology, Center for Pollinator Research, Huck Institutes of the Life Sciences, Pennsylvania State University, University Park, PA 16803, USA; advaudo@gmail.com (A.D.V.); christina.grozinger@gmail.com (C.M.G.); 4 Department of Biology, University of Nevada Reno, Reno, NV 89557, USA

**Keywords:** mutualisms, pollinators, bees, climate change, experimental warming, *Carduus nutans*, pollen nutrition

## Abstract

Climate warming is likely to change the ways in which plants interact with their insect mutualists, for example through changes in phytochemistry. In particular, this may have implications for the ways in which we manage noxious weeds, which may spread more quickly if they experience stronger mutualistic interactions. We grew the invasive nodding thistle, *Carduus nutans*, in two experimental treatments in the field: either passively warmed with open top chambers or at ambient temperatures. We collected pollen from thistles in each treatment and analysed the total protein, lipid, and carbohydrate content. We observed no difference in the pollen protein or carbohydrate content, but the total lipid content of the pollen was significantly higher in warmed plants. We conducted a total of 12.75 h of observations of putatively mutualistic, flower-visiting insects. In addition, we spent 4.17 h collecting bees that visited thistle inflorescences in the treatments, allowing us to identify them to species. We found a significant increase in the abundance of flower-visiting insects in the observations, but not bee abundance in collections. In addition, there was no treatment effect on the number of flower-visiting morphotypes in the observations, or bee species richness in the collections. However, a nonparametric test did identify a significant effect of warming on the composition of flower-visiting morphotypes in observations and bee species in collections. Overall, the warming treatment significantly increased lipid content of the pollen, but had relatively weak effects on insect visitation patterns. However, these effects may be amplified at larger spatial and temporal scales or higher temperatures.

## 1. Introduction

Many biological responses to climate change have been documented, including range shifts [1], changes in plant growth [2] and dispersal [3,4], and changes in floral resource provisioning [5,6]. In plant invasions, all of these attributes are also related to the success of invasions into novel habitats. For example, non-native plant species may attract pollinating insects in novel ecosystems if the quality of their pollen is high relative to coflowering plant species [7]. It is important to better understand how a changing climate will affect the invasive potential of noxious weeds through changes in plant traits and consequently their attractiveness to mutualists.

The mechanisms behind altered plant–insect interactions resulting from global change are still not well understood. There is a growing body of research on how warming temperatures might change interactions between plants and insect herbivores through changes in their phytochemistry [8] or mechanical defences [9], but the effects on interactions are context-dependent [10]. Moreover, research suggests that the quality of floral resources will change with a changing climate [5]. Changes in nectar production have been documented, with nectar production increasing with temperature to a point [11] and decreasing in other cases [6,12]. Similarly, though the effects of warming on pollen are not well-established, there is some evidence indicating that plants may produce less pollen [13], or less viable pollen [14] at higher temperatures. Though research has established that pollen nutritional quality (e.g., the concentration of macronutrients) can change with plant health [15], it is less clear how this nutritional quality will change in response to increasing temperature.

We applied an experimental warming treatment to quantify the impact of passive warming on the pollen nutrition and pollinator visitation of a noxious invader, *Carduus nutans* (the nodding thistle), using open top chambers (OTCs). We expected to see a change in the nutritional quality of the thistle and a resulting change in the flower visitation by pollinating insects.

## 2. Methods

### 2.1. Study Species

*C. nutans* is a thistle invasive in the Americas, Australia, and New Zealand [16]. In North America, *C. nutans* is one of the top ten most noxious weeds in agricultural systems [17]. Previous research has shown that climate warming has the potential to change the growth of the plant [2], timing of its flowering [2], potential spread of its wind-dispersed seeds [3,4], and its mechanical defences [9]. Outcrossing pollination in this thistle species is accomplished through flower-visiting insects (putative pollinators), which are also important for mediating competition with congeneric thistles [18].

### 2.2. Experimental Set-Up

Because *C. nutans* is a monocarpic perennial with size-dependent flowering, we grew thistles from seed the previous year in greenhouses and then planted them in the experimental plots the autumn before the sampling was conducted. We used open top chambers (OTCs) to passively warm the treated plots; OTCs are plastic cones used for in situ warming of small (in this case 2 m × 2 m) plots and are 35–40 cm tall [19]. These open top chambers often warm median daily temperatures by approximately 1.5 °C [20]. The OTCs in our experiment were in place from when the thistle rosettes were planted in the field until 18 December 2015. They were then removed, to avoid confounding snow cover duration and temperature effects. Chambers were reinstalled on 4 April 2016 and remained in place until the plants were harvested at the end of the season. There were 32 2 m × 2 m research plots in 8 blocks, with four *C. nutans* rosettes in each plot. Each block had two OTC plots and two ambient (control) plots.

### 2.3. Pollen Collection and Analysis

To determine whether the nutrition provided by the warmed plants changed in its macronutrient (protein, lipid, carbohydrate) content, we collected fresh pollen from flowering thistles in the two treatments. The pollen was gently brushed off mature inflorescences where the anthers had dehisced. We pooled pollen from multiple inflorescences and plants in each treatment across the summer to obtain enough pollen to conduct the analysis. All pollen was stored at −20 °C until we could conduct the analysis and then we randomly subsampled the mixed pollen three times to calculate the error of our measures. The protein concentration of the pollen was measured using a Bradford assay, while the lipid and carbohydrate concentrations were measured using an assay modified from Van Handel and Day [21,22]. We measured all concentrations (lipid, protein, carbohydrate) in micrograms per milligram of pollen. The full description of the pollen nutritional methodology is available in Vaudo et al. [22].

### 2.4. Observations

We conducted pollinator observations by identifying flower-visiting insect morphotypes that could be distinguished by eye in the field. We conducted roughly 12.75 h of pollinator observations during the flowering season. On each observation date, we spent 2.5 min observing any insect visitors to the thistle inflorescences of each plot. We conducted flower-visitor observations on all plots where a thistle was in flower on each sample date, and kept records of the number of inflorescences in bloom in each plot during the observations. We recorded insect flower visitors according to the following functional categories: honey bees (HB), bumble bees (BB), large carpenter bees (LCB), large dark bees (LDB), small dark bees (LDB), green sweat bees, (GSB), hairy leg bees (HLB), dark hairy belly bees (DHB), flower flies, other flies, other bees, Lepidoptera, soldier beetles (*Chauliognathus pensylvanicus*), and “other”.

### 2.5. Collections

We conducted 4.17 h of bee collections using an insect vacuum at the plots. On each sampling day, we spent 2.5 min at each research plot where the thistles were flowering and collected all bee visitors to the inflorescences of the thistles. We kept the insect vacuum off until a bee visitor made contact with the reproductive parts of the inflorescence, then we turned it on briefly to collect the visitor before turning it off again. We did not notice any change in visitor behaviour with the noise of the vacuum. We also recorded the number of thistle inflorescences in flower in each plot on each sampling day to control for the effect of floral display on pollinator visitation. The bees were then pinned, labelled, and identified with the help of Samuel Droege of the United States Geological Survey (USGS).

### 2.6. Data Analysis

First, we tested whether there was a significant difference in the temperature of warmed and ambient plots using a general linear mixed effects models (GLMM, R package “lme4” [23]). We also used GLMMs to determine whether the pollen collected from warmed or ambient thistles differed significantly from one another in protein, lipid, or carbohydrate content. Note that the variation measured here is in the subsamples taken from the pooled pollen from each treatment and not from the experimental blocks. This is common in pollen nutrition methods as it is challenging to collect enough pollen for the analysis from each block.

Next, we tested for a correlation between the number of inflorescences and the number of visitors in a given observation or collection, using a Pearson correlation coefficient. We then used GLMMs to determine whether there were differences in the number of inflorescences produced in the two different treatments. Where the data were non-normal, we tested for overdispersion of the model, and when the overdispersion was significant, we used a Laplace approximation (Poisson distribution, log link). Where the Laplace approximation was not sufficient to resolve the overdispersion, we log transformed the data. We built regressions for the following response variables: inflorescence number, abundance and number of morphotypes in the observations, and abundance and species richness of the collected bees. For each full model, we included treatment (ambient vs. OTC) as a fixed effect; for the insect collections/observations, we also included the number of inflorescences as a fixed effect, and tested for an interaction effect. The random effects in this analysis were the block nested within the sample date, to account for repeated measures, and observer identity. For the collected bee specimens, we also conducted a rarefaction analysis to evaluate sample completeness and species diversity (R package “iNEXT” [24]).

Finally, we used Chi-squared tests to determine whether the warming treatment had an effect on the relative abundances of flower-visitor morphotypes in the observations ambient and warmed plots and bee species in the collections. The Chi-squared tests are nonparametric, allowing a comparison of raw abundances rather than log-transformed abundances. We also used Chi-squared tests to compare our different methods of collecting data on pollinating insects; testing whether the abundance of observed pollinator morphotypes differed from the collected bee species (retroactively sorted into the same morphotype categories). For these Chi-squared tests, we combined all categories (morphotypes or species) that summed to less than 5 into a category we called “other”.

## 3. Results

### 3.1. Treatment Effects on Plants

**Figure 1:**
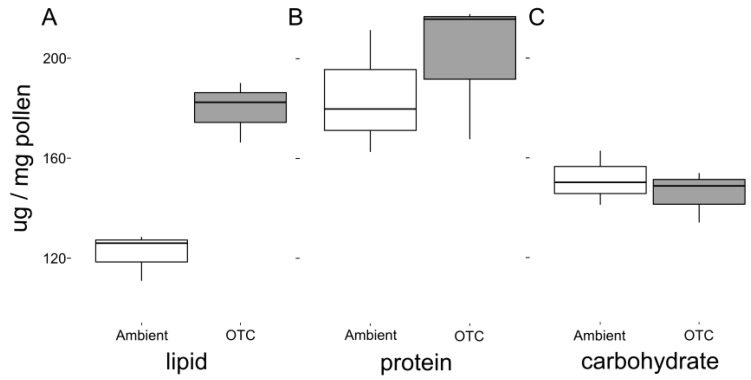
Boxplots comparing the concentration (ug/mg pollen) of (**A**) lipid, (**B**) protein, and (**C**) carbohydrate from subsamples of pooled collections from ambient and warmed (OTC) plots.

On average, the OTCs warmed the plots by 0.48 °C relative to the ambient plots (df = 30.12, *t*-value = 4.11, *p* < 0.001). There was no significant difference in the number of inflorescences produced in control (ambient) vs. warmed (OTC) plots (Table 1). There was no significant difference between the pollen protein or carbohydrate content in ambient vs. warmed plants, but warmed plants had, on average, a 38% higher pollen lipid concentration (Table 1, Figure 1).

### 3.2. Observed Flower-Visiting Insects

We observed a total of 1351 insect visitors on 2587 inflorescences. We observed 14 different morphotypes, the most abundant of which were, in order of decreasing abundance, soldier beetles (*C. pensylvanicus*), bumble bees (*Bombus* spp.), and flies (other than flower flies). There was not a significant effect of the warming treatment on the number of inflorescences recorded during the observations (Table 1). However, there was a significant correlation between the number of inflorescences and the number of insect visitors in a given sample (Pearson correlation coefficient = 0.40, *p* << 0.001). There was also a significant positive association between the abundance of observed insects (log transformed) and the number of inflorescences, as well as a positive effect of the warming treatment on the abundance of flower-visiting insects (Table 1). A Chi-squared test also showed a significant difference between the composition of the observed flower-visitors in ambient and warmed plots (Chi-squared statistic = 25.44, df = 12, *p* = 0.01). The largest differences in morphotype composition were a lower abundance of small dark bees and a larger abundance of bumble bees and soldier beetles in warmed plots. There was no significant effect of the warming treatment on bee visitors alone nor on just non-bee visitors alone in the observations (Table S1).

### 3.3. Collected Bees

**Figure 2:**
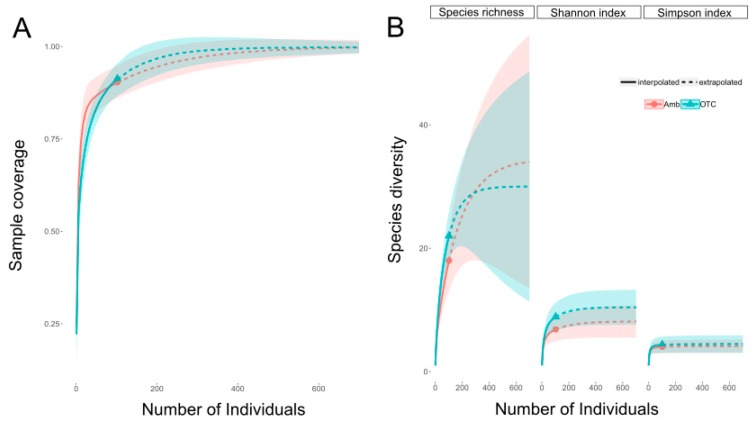
Sample coverage of the expected (**A**) bee species richness and (**B**) diversity rarefaction for the collected bee specimens in ambient (red) and warmed (OTC, blue) treatments. The shaded regions indicate 95% confidence intervals.

We collected a total of 204 bee specimens, representing 27 bee species (Table S2). Using a rarefaction analysis, we calculated our expected sample coverage as 90.3% of the expected bee species richness for ambient and 91.3% for OTC plots (Figure 2). The most abundant species was *Bombus bimaculatus*, followed by *Halictus ligatus* and *Melissodes desponsa*. There was not a significant effect of warming on the number of inflorescences counted during collections (Table 1), but there was a significant correlation between the number of inflorescences and the number of bee visitors in a given sample (Pearson correlation coefficient = 0.33, *p* < 0.001). In addition, the number of inflorescences was a significant predictor of bee abundance in the plots, but inflorescences did not predict bee species richness (Table 1). There was not a significant relationship between the warming treatment and the abundance or richness of collected pollinating insects. Moreover, a rarefaction analysis showed overlapping confidence intervals between the treatments for species richness, as well as Shannon and Simpson diversity indices (Figure 2). However, a Chi-squared test showed a significant difference in the bee species composition in ambient and warmed plots (Chi-squared statistic = 14.96, df = 7, *p* = 0.04). The largest differences between the relative abundance of bee species were a greater abundance of uncommon bees (fewer than 5 specimens per species pooled into a common category for the Chi-squared test) and a lower abundance of *B. perplexus* in the warmed plots.

**Figure 3:**
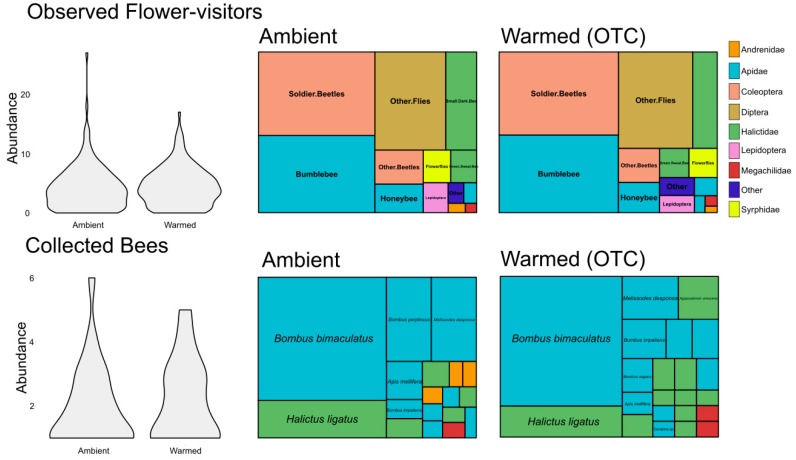
Violin plots and treemaps showing the relative abundances and composition of different morphotypes in observed flower-visiting insects (**top**) and the species of collected bees (**bottom**), as well as a comparison of the ambient (**left**) and warmed (**right**) treatments. The violin plots illustrate the non-normal distributions of the abundance data. Colours refer to the order or family of the insects.

We compared observed flower-visitors to collected bee species, to determine the difference in our data collection methodologies. One fundamental difference between the observations and collections was that observations recorded non-bee morphotypes, while the collections included only bees. The observations demonstrated the high relative abundance of non-bee visitors, primarily pollen predators such as the soldier beetles (Figure 3). We focused on bees for our collections because they are often considered to be an important group of pollinating insects. Because we also recorded bee morphotypes in our observations, we compared the observed bee morphotypes to the collected bees, and found significant differences in the distributions overall (chi squared statistic = 39.55, df = 7, *p* < 0.001). The biggest differences were that there were more honey bees in the observations and more hairy leg bees (*Melissodes* spp.) in the collections (Figure S1). Though these distributions were significantly different, the observers recorded all of the morphotypes represented in the collections, and the proportions were fairly similar.

We also used a Chi-squared test to compare warmed and ambient plots in observations and collections. However, this test identified contradictory effects on particular groups between the observations and collections. For example, bumble bees were more common in warmed plots in the observations, but there were fewer bumble bees (especially *B. perplexus*) in warmed plots in the collections. Similarly, there were many fewer small dark bees in the observations, but there were more *Lasioglossum* (the genus contributing most to the small dark bee morphotype) specimens in warmed plots in the collections.

## 4. Discussion

We measured the nutritional quality of an invasive thistle’s pollen (protein, lipid, and carbohydrate content) to determine whether it responded to an experimental passive warming treatment, and quantified flower-visitation by insects (putative pollinators) to determine whether they responded to changes in this pollen nutrition. We quantified a significant increase in the pollen lipid concentration (but not protein or carbohydrate concentration) of warmed thistles. The thistles in the warmed treatment had small significant increases in the abundance of observed flower-visiting insects and a significant difference in the composition of morphotypes in observations and species in collections. However, there was no effect of the treatment on the abundance of collected bees and no differences in the number of flower-visiting morphotypes or species diversity of collected bees. Similarly, the treatment did not have a significant effect on the observed flower-visitors when they were separated into separate bee and non-bee categories. These significant effects are compelling, given the relatively minor warming of the OTCs (−0.5 °C) and the close spatial proximity of the plants in the ambient and warmed treatments, and prompt additional questions about the effects of further warming on pollen macronutrient concentrations and on the related insect communities.

We recorded a positive effect of warming on the pollen lipid concentration. The absence of a strong, consistent effect on the abundance of bee visitors in response was surprising because evidence suggests that pollen-feeding insects are able to detect changes in floral resource quality and plant health, and change their foraging behaviour accordingly, even within the same season [15,25]. In particular, there is evidence that bees can detect pollen quality [22,26], and may exhibit preferences for certain nutritional attributes [7]. However, the role of lipid concentration in pollinator nutrition is unclear, as the quality of pollen is sometimes considered to be related to protein content, and lipid content may vary less than protein content ([27] but see [22]). In addition, though it seems that lipids are important for honey bee health [28] (e.g., in developing fat bodies [27]), it has also been shown that bumble bees regulate the relative concentrations of lipids and proteins in their pollen provisions [22]. Thus, the observed increase in lipid concentration may not have a clear benefit or detriment to floral visitors. On the other hand, though the effects of warming on pollen are not well-established, there is evidence indicating that plants may produce less pollen [13], or less viable pollen [14] at higher temperatures, attributes which we did not quantify here.

We also did not measure changes in nectar production or quality in this study, but these traits have also been shown to respond to warming temperatures. For example, several studies have demonstrated concurrent changes in nectar quality and bee visitation in response to temperature, though there is no consensus on the magnitude or directionality of these effects [5]. On the other hand, one study showed changes in nectar quantity in response to passive warming treatments, but also did not show changes in flower-visitor interactions [6]. It would be interesting to see whether there were changes in nectar production in thistles grown in warmed conditions, despite few changes in insect interactions.

It is important to note that we saw significant differences in our two methods of quantifying flower-visitor interactions (collections and observations), in that the observations detected more honey bees and fewer *Melissodes* spp. This difference could be due to misidentification in observations, where determinations have to be made quickly, or due to background stochasticity in their abundances. However, the difference between the two methods was relatively minor, and all collected bees fit well into the morphotypes recorded in the observations. On the other hand, though there were significant differences in the compositions of both observed flower-visitors and collected bees in warmed and ambient plots, these differences contradicted one another. In collections, bumble bees were less common in warmed plots, while in observations they were more common in warmed plots. Similarly, there were more *Lasioglossum* specimens in collections from warmed plots, but fewer small dark bees from observations in warmed plots. This could indicate either that observing flower-visitors is more likely to lead to misleading answers due to misidentification, or that there were not strong, consistent effects of passive warming on the composition of the bee community.

## 5. Conclusions

A changing climate is likely to change the interactions between invasive species and their mutualists. We showed that even a small degree of passive experimental warming resulted in a significant increase in the lipid concentration of the invasive thistle’s pollen. Though we detected only minor changes in the thistle’s interactions with flower-visiting insects, the accumulation of small effects may have implications for the management of this noxious weed. Similar warming effects may pertain for other invasive and endangered species, leading to changes in species interactions that have significant implications for fitness of both the plant host and the insect; shifts in insect nutrition available in future climates will clearly be an important topic of research in the years to come.

## Figures and Tables

**Table 1:** General linear mixed effects models (GLMMs) used to test for the effects of treatment, observer type, and inflorescence number, with significant values in bold.

Group	Response Variable	Distribution	Comparison	Variable Type	Effect SIZE	z- or *t*-Value	*p*-Value
**Observed flower-visitors**	Inflorescences	*poisson*(*link* = “*log*”)	OTC-Ambient	categorical	−0.08	−0.7	0.48
	log transformed Abundance	*gaussian*(*link* = “*identity*”)	Inflorescences	continuous	0.05	7.68	**<<0.001**
		*gaussian*(*link* = “*identity*”)	OTC-Ambient	categorical	0.15	2.25	**0.02**
	Morphotypes	*poisson*(*link* = “*log*”)	Inflorescences	continuous	0.03	5.07	**<<0.001**
		*poisson*(*link* = “*log*”)	OTC-Ambient	categorical	0.07	0.97	0.33
**Collected bees**	Inflorescences	*poisson*(*link* = “*log*”)	OTC-Ambient	categorical	−0.13	−1.08	0.28
	Abundance	*poisson*(*link* = “*log*”)	Inflorescences	continuous	0.02	2.18	**0.03**
		*poisson*(*link* = “*log*”)	OTC-Ambient	categorical	0.15	1.08	0.28
	Richness	*gaussian*(*link* = “*identity*”)	Inflorescences	continuous	0.01	1.2	0.23
		*gaussian*(*link* = “*identity*”)	OTC-Ambient	categorical	0.15	0.96	0.34
**Pollen nutrition**	Lipid concentration	*gaussian*(*link* = “*identity*”)	OTC-Ambient	categorical	57.76	6.48	**0.003**
	Carbohydrate concentration	*gaussian*(*link* = “*identity*”)	OTC-Ambient	categorical	−5.84	−0.68	0.54
	Protein concentration	*gaussian*(*link* = “*identity*”)	OTC-Ambient	categorical	15.8	0.73	0.51

## Supplementary Materials

The following are available online at https://www.mdpi.com/2075-4450/11/1/20/s1, Figure S1: Treemaps showing the relative abundances of different morphotypes in observed flower-visiting insects (A) and the species of collected bees (B), as well as a comparison of the observed counts of bee morphotypes in collections and observations (C) with the expected counts if there were no difference between observations and collections (D). Colours refer to the order or family of the insects. Table S1: Results of additional GLMMs. Table S2: Bee species collected on *C. nutans.*

## References

[1] Chen I.C., Hill J.K., Ohlemüller R., Roy D.B., Thomas C.D. (2011). Rapid range shifts of species associated with high levels of climate warming. Science.

[2] Zhang R., Post E., Shea K. (2012). Warming leads to divergent responses but similarly improved performance of two invasive thistles. Popul. Ecol..

[3] Teller B.J., Zhang R., Shea K. (2016). Seed release in a changing climate: Initiation of movement increases spread of an invasive species under simulated climate warming. Divers. Distrib..

[4] Zhang R., Jongejans E., Shea K. (2011). Warming increases the spread of an invasive thistle. PLoS ONE.

[5] Scaven V., Rafferty N. (2013). Physiological effects of climate warming on flowering plants and insect pollinators and potential consequences for their interactions. Curr. Zool..

[6] Mu J., Peng Y., Xi X., Wu X., Li G., Niklas K.J., Sun S. (2015). Artificial asymmetric warming reduces nectar yield in a Tibetan alpine species of Asteraceae. Ann. Bot..

[7] Russo L., Vaudo A.D., Fisher C.J., Grozinger C.M., Shea K. (2019). Bee community preference for an invasive thistle associated with higher pollen protein content. Oecologia.

[8] Jamieson M.A., Burkle L.A., Manson J.S., Runyon J.B., Trowbridge A.M., Zientek J. (2017). Global change effects on plant–insect interactions: The role of phytochemistry. Curr. Opin. Insect Sci..

[9] Zhang R., Leshak A., Shea K. (2012). Decreased structural defence of an invasive thistle under warming. Plant Biol..

[10] Roy B.A., Güsewell S., Harte J. (2004). Response of plant pathogens and herbivores to a warming experiment. Ecology.

[11] Petanidou T., Smets E. (1996). Does temperature stress induce nectar secretion in Mediterranean plants?. New Phytol..

[12] Takkis K., Tscheulin T., Tsalkatis P., Petanidou T. (2015). Climate change reduces nectar secretion in two common Mediterranean plants. AoB Plants.

[13] Koti S., Reddy K.R., Reddy V.R., Kakani V.G., Zhao D. (2005). Interactive effects of carbon dioxide, temperature, and ultraviolet-B radiation on soybean (*Glycine max* L.) flower and pollen morphology, pollen production, germination, and tube lengths. J. Exp. Bot..

[14] Hodge S., Prasad A. (2013). Factors influencing the foraging activity of the allodapine bee *Braunsapis puangensis* on creeping daisy (*Sphagneticola trilobata*) in Fiji. J. Hymenopt. Res..

[15] Cardoza Y.J., Harris G.K., Grozinger C.M. (2012). Effects of soil quality enhancement on pollinator-plant interactions. Psyche J. Entomol..

[16] Jongejans E., Sheppard A.W., Shea K. (2006). What controls the population dynamics of the invasive thistle *Carduus nutans* in its native range?. J. Appl. Ecol..

[17] Skinner K., Smith L., Rice P. (2000). Using noxious weed lists to prioritize targets for developing weed management strategies. Weed Sci..

[18] Yang S., Ferrari M.J., Shea K. (2011). Pollinator behavior mediates negative interactions between two congeneric invasive plant species. Am. Nat..

[19] Hollister R.D., Webber P.J. (2000). Biotic validation of small open-top chambers in a tundra ecosystem. Glob. Chang. Biol..

[20] Yang Y., Halbritter A.H., Klanderud K., Telford R.J., Wang G., Vandvik V. (2018). Transplants, open top chambers (OTCs) and gradient studies ask different questions in climate change effects studies. Front. Plant Sci..

[21] Van Handel E., Day J.F. (1988). Assay of lipids, glycogen and sugars in individual mosquitoes: Correlations with wing length in field-collected *Aedes vexans*. J. Am. Mosq. Control Assoc..

[22] Vaudo A.D., Patch H.M., Mortensen D.A., Tooker J.F., Grozinger C.M. (2016). Macronutrient ratios in pollen shape bumble bee (*Bombus impatiens*) foraging strategies and floral preferences. Proc. Natl. Acad. Sci. USA.

[23] Bates D., Mächler M., Bolker B., Walker S. (2014). Fitting linear mixed-effects models using lme4. J. Stat. Softw..

[24] Chao A., Gotelli N.J., Hsieh T.C., Sander E.L., Ma K.H., Colwell R.K., Ellison A.M. (2014). Rarefaction and extrapolation with Hill numbers: A framework for sampling and estimation in species diversity studies. Ecol. Monogr..

[25] Russo L., Shea K. (2017). Experimentally increased network connectance is associated with increased bee species richness and abundance in a plant-pollinator community. J. Complex Netw..

[26] Vaudo A.D., Stabler D., Patch H.M., Tooker J.F., Grozinger C.M., Wright G.A. (2016). Bumble bees regulate their intake of the essential protein and lipid pollen macronutrients. J. Exp. Biol..

[27] Di Pasquale G., Salignon M., Le Conte Y., Belzunces L.P., Decourtye A., Kretzschmar A., Suchail S., Brunet J.L., Alaux C. (2013). Influence of pollen nutrition on honey bee health: Do pollen quality and diversity matter?. PLoS ONE.

[28] Brodschneider R., Crailsheim K. (2010). Nutrition and health in honey bees. Apidologie.

