# Supplementary material for: Warming Increases Pollen Lipid Concentration in an Invasive Thistle, with Minor Effects on the Associated Floral-Visitor Community
Source: Insects. 2019 Dec 25;11(1):20. doi: 10.3390/insects11010020 (PMC7022432; doi:10.3390/insects11010020)
Supplement: Supplementary file 1 [file insects-11-00020-s001.pdf]

Supplemental Materials

Table 1. Results of additional GLMMs.

Group	Response variable	Distribution	Comparison	Variable type	Effect Size	z or t value	P value
**Observed non-bee flower-visitors**	log transformed Abundance	*gaussian(link="identity")*	OTC - Ambient	categorical	0.09	1.19	0.24
		gaussian(link="identity")	Inflorescences	continuous	0.04	6.18	**<< 0.001**
**Observed bee flower-visitors**	log transformed Abundance	*gaussian(link="identity")*	OTC - Ambient	categorical	0.08	1.18	0.24
		gaussian(link="identity")	Inflorescences	continuous	0.04	8.43	**<< 0.001**

Table 2. Bee species collected on *C. nutans*.

Family	Morphotype	Species	Count
Halictidae	Green Sweat Bee	*Agapostemon virescens*	7
Andrenidae	Large Dark Bee	*Andrena crataegi*	1
Andrenidae	Large Dark Bee	*Andrena nuda*	1
Andrenidae	Large Dark Bee	*Andrena perplexa*	1
Apidae	Honeybee	*Apis mellifera*	11
Halictidae	Green Sweat Bee	*Augochlorella aurata*	1
Apidae	Bumblebee	*Bombus bimaculatus*	132
Apidae	Bumblebee	*Bombus griseocollis*	5
Apidae	Bumblebee	*Bombus impatiens*	8
Apidae	Bumblebee	*Bombus perplexus*	19
Apidae	Bumblebee	*Bombus terricola*	1
Apidae	Bumblebee	*Bombus vagans*	5
Apidae	Small Dark Bee	*Ceratina calcarata*	1
Apidae	Small Dark Bee	*Ceratina sp.*	1
Halictidae	Small Dark Bee	*Halictus confusus*	1
Halictidae	Small Dark Bee	*Halictus ligatus*	28
Halictidae	Small Dark Bee	*Halictus rubicundus*	2
Halictidae	Small Dark Bee	*Lasioglossum hitchensi*	5
Halictidae	Small Dark Bee	*Lasioglossum leucozonium*	2
Halictidae	Small Dark Bee	*Lasioglossum oceanicum*	1
Halictidae	Small Dark Bee	*Lasioglossum paradmirandum*	4
Halictidae	Small Dark Bee	*Lasioglossum pectorale*	2
Halictidae	Small Dark Bee	*Lasioglossum trigeminum*	1
Halictidae	Small Dark Bee	*Lasioglossum versatum*	4
Megachilidae	Dark Hairy Belly Bee	*Megachile pugnata*	2
Megachilidae	Dark Hairy Belly Bee	*Megachile relativa*	1
Megachilidae	Dark Hairy Belly Bee	*Megachile rotundata*	1
Apidae	Hairy Leg Bee	*Melissodes desponsa*	23
Apidae	Large Carpenter Bee	*Xylocopa virginica*	3

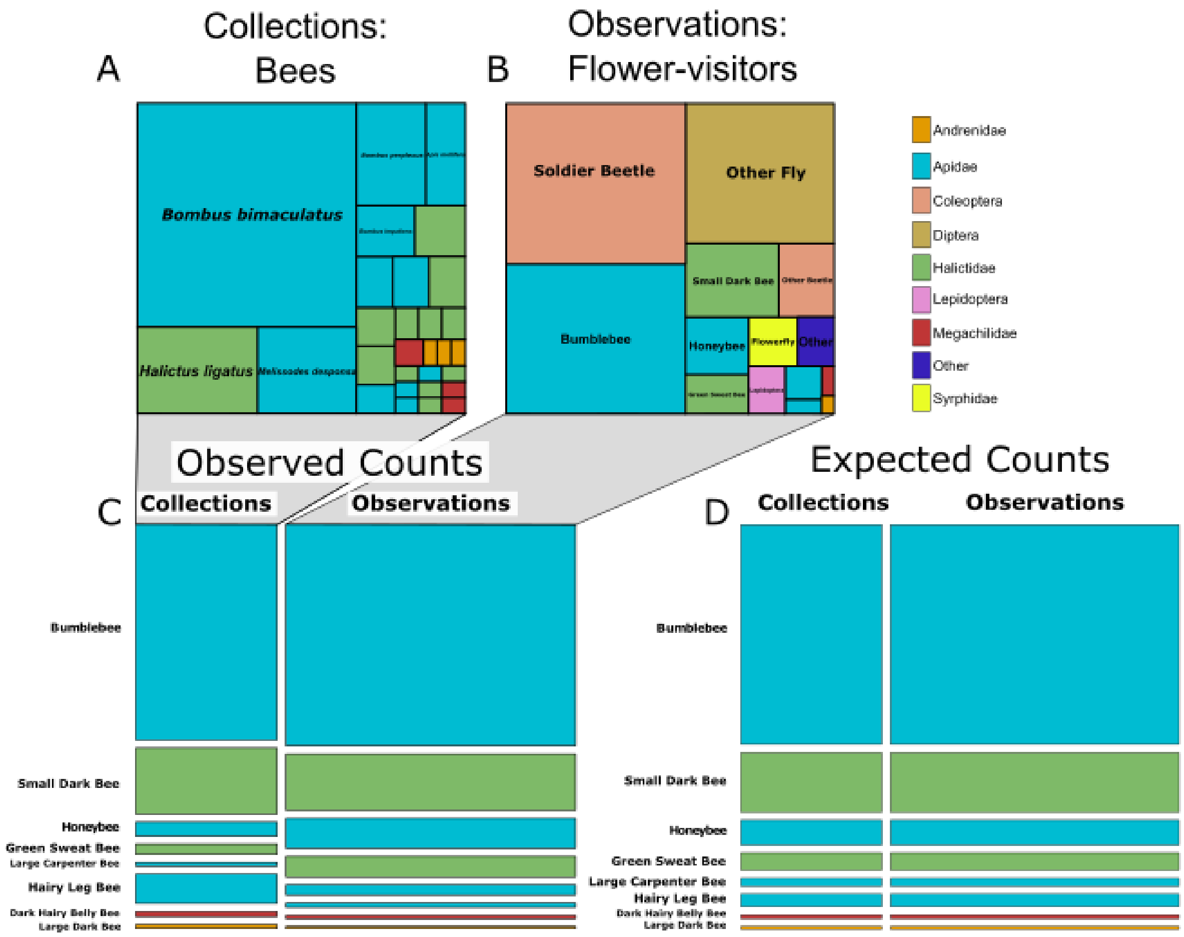

Figure 1. Treemaps showing the relative abundances of different morphotypes in observed flower-visiting insects (A) and the species of collected bees (B), as well as a comparison of the observed counts of bee morphotypes in collections and observations (C) with the expected counts if there were no difference between observations and collections (D). Colours refer to the order or family of the insects.
